# Identifying essential proteins from protein–protein interaction networks based on influence maximization

**DOI:** 10.1186/s12859-022-04874-w

**Published:** 2022-08-16

**Authors:** Weixia Xu, Yunfeng Dong, Jihong Guan, Shuigeng Zhou

**Affiliations:** 1 School of Information Management, Shanghai Lixin University of Accounting and Finance, Shanghai, China; 2 Shanghai Key Lab of Intelligent Information Processing, and School of Computer Science, Fudan University, Shanghai, China; 3 Department of Computer Science and Technology, Tongji University, Shanghai, China

**Keywords:** Protein–protein interaction network, Essential proteins, Influence maximization, Influence discount

## Abstract

****Background**:**

Essential proteins are indispensable to the development and survival of cells. The identification of essential proteins not only is helpful for the understanding of the minimal requirements for cell survival, but also has practical significance in disease diagnosis, drug design and medical treatment. With the rapidly amassing of protein–protein interaction (PPI) data, computationally identifying essential proteins from protein–protein interaction networks (PINs) becomes more and more popular. Up to now, a number of various approaches for essential protein identification based on PINs have been developed.

****Results**:**

In this paper, we propose a new and effective approach called iMEPP to identify essential proteins from PINs by fusing multiple types of biological data and applying the influence maximization mechanism to the PINs. Concretely, we first integrate PPI data, gene expression data and Gene Ontology to construct weighted PINs, to alleviate the impact of high false-positives in the raw PPI data. Then, we define the *influence scores* of nodes in PINs with both orthological data and PIN topological information. Finally, we develop an influence discount algorithm to identify essential proteins based on the influence maximization mechanism.

****Conclusions**:**

We applied our method to identifying essential proteins from *saccharomyces cerevisiae* PIN. Experiments show that our iMEPP method outperforms the existing methods, which validates its effectiveness and advantage.

## Background

Proteins [1, 2] are important structural and functional components of cells, they play many critical functions of living organisms, including carrier transport, antibody immunity, hormone regulation and so on. Among all, essential proteins are those indispensable to the development and survival of cells. It was also shown that the pathogenic genes are closely related to the essential proteins. Therefore, the identification of essential proteins not only is helpful for the understanding of the minimal requirements for cell survival, but also has great practical significance for the study of pathogenic biology [3] and drug design [4].

Wet lab experiments are firstly used to identify essential proteins, including single gene knockouts [5], RNA interference and anti-sense RNA [6] etc. Though these methods are very accurate, they are expensive and time-consuming. With the rapid development of high-throughput experimental technology, it is very convenient to obtain large amounts of protein-protein interaction (PPI) data. This inspires the development of computational methods [7–9] to identify essential proteins. Most existing computational methods are based on PPI networks (PINs), which are graphic representations of PPI data. A PIN can be modeled as a graph denoted by *G*(*E*, *V*), where *V* is the set of nodes representing the proteins, and *E* is the set of edges representing the interactions between the proteins. From graph theory perspective, essential proteins can be seen as the important or key nodes in a PIN. So essential protein identification turns to finding important nodes in a PIN.

Jeong et al. [10] proposed the *centrality-lethality* rule, which indicates that essential proteins tend to be more important to the survival of cells than the other proteins. Thus, the deletion of essential proteins is more lethal than the deletion of the other proteins. Based on the *centrality-lethality* rule, various centrality measures are proposed to identify essential proteins, including degree centrality (DC) [10], betweenness centrality (BC) [11], closeness centrality (CC) [12], subgraph centrality (SC) [13]), and eigenvector centrality (EC) [14] etc.

Following that, more sophisticated metrics that exploit deep topological information of PINs have also been proposed to identify essential proteins from PINs, which can achieve better performance than the centrality based methods. Furthermore, considering of high false-positives in PINs, some methods use additional biological data to boost performance. Li et al. proposed the PeC [15] algorithm by combining gene expression data and the topological information of PINs. Zhang et al. developed the CoEWc [16] method that uses local clustering coefficient and Pearson correlation coefficient (PCC) of gene expression data. Later, Zhang et al. introduced the TEO [17] method to integrate gene expression data, Gene Ontology (GO) and orthology data for essential protein identification. Recently, Xu et al. [9] proposed a random walk based method EssRank that exploits gene expression data, functional annotations, domain interactions and phylogenetic profiles to improve the quality of PINs and subsequently to achieve better identification accuracy.

In this paper, inspired by the influence maximization (IM) mechanism in social networks for viral marketing, we propose a novel method called iMEPP to identify essential proteins from PINs. On the one hand, we use PPI data, gene expression data and GO to construct weighted PINs for reducing the impact of high false-positives in raw PPI data. On the other hand, we adapt the IM mechanism in social networks to the essential protein identification problem. To this end, we define the *influence scores* (IS) of nodes in PINs with both orthological data and PIN topological information, and develop an influence discount (ID) algorithm to identify essential proteins from PINs. Our experiments on *saccharomyces cerevisiae* data show that the proposed iMEPP method can achieve better performance than the existing methods.

## Results

In this section, we first introduce the PPI data and gene expression data of *saccharomyces cerevisiae*. Then, we give the experimental settings. Finally, the experimental results are reported.

### Datasets

PPI data and gene expression data of *saccharomyces cerevisiae* are used in our experiments. PPI data come from the BioGRID database [18], including 4860 proteins and 22138 interactions between proteins. Essential protein data are collected from the SGD [19], DEG [20] and SGDP [21] databases, totally 1194 essential proteins. Orthology data are from the InParanoid (version 7) database [22], containing 100 genomes where 99 are eukaryotes and 1 is prokaryote.

### Experimental settings

$\lambda$ is a tradeoff parameter to balance the the contribution of topology and orthology. When $\lambda =0$, the identification of essential proteins is totally determined by the influence of PIN topology; and if $\lambda =1$, it is only determined by protein orthology. By setting $p = 0.001$ [23] and the value of $\lambda$ to 0, 0.1, 0.2, ..., 1 respectively, we check the number of essential proteins correctly identified by our method.

To show the advantage of our method, we compare it with several existing methods, including five centrality based methods (BC [11], CC [12], DC [10] and EC [14], SC [13]), three methods integrating multiple types of biological information (PeC [15], CoEWc [16] and TEO [17]). Furthermore, we also implement another influence maximization algorithm degree discount (DD) [24] for comparison. We let each method output top-*k* (*k* is taken from 100 to 1000) essential protein candidates, from which we count the number of correctly identified ones.

### Experimental results

#### The impact of $\lambda$

Table 1 gives the numbers of correctly identified essential proteins for different $\lambda$ and *k* values. We set *k* from 100 to 600, and for each *k* value, we increase $\lambda$ from 0 to 1.0. From Table 1, we can see that given the *k* value, neither $\lambda=0$ nor $\lambda=1.0$ can get the best result. This means that combining PIN topology and protein orthology is beneficial to essential protein identification. When $\lambda$ falls between 0.2 and 0.5, we can get better result. This indicates that PIN topology is more important than protein orthology in essential protein identification. Furthermore, in most cases we get the best result when $\lambda=0.2$, so in the remaining experiments we set $\lambda=0.2$ in our method.

**Table 1:** The numbers of correctly identified essential proteins for different $\lambda$ and *k* values

k\λ	0	0.1	0.2	0.3	0.4	0.5	0.6	0.7	0.8	0.9	1
100	72	83	85	86	88	**89**	88	83	80	75	68
200	133	162	**168**	**168**	162	154	152	146	143	139	133
300	192	229	**236**	228	218	219	215	209	204	197	192
400	240	279	**285**	282	280	273	272	271	266	264	251
500	278	333	**337**	332	327	325	322	314	311	309	307
600	317	381	**382**	375	370	367	364	361	359	358	350

Each bold number in the table indicates the largest number of identified essential proteins for a given *k* value

#### Comparison with existing methods

**Fig. 1:**
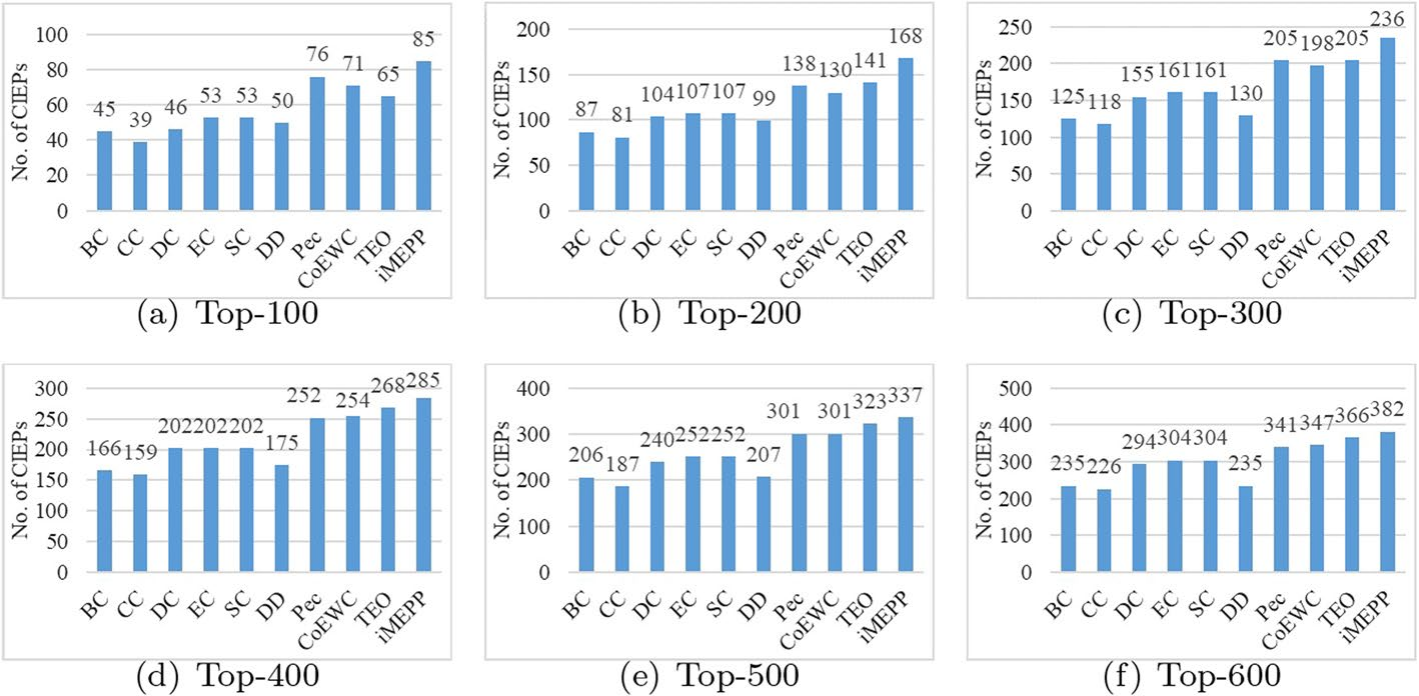
Comparison results when top-*k* (*k* is from 100 to 600) candidates are output

First, we examine the top 100, 200, 300, 400, 500, 600 output candidates respectively, and count the corresponding numbers of correctly identified essential proteins. The comparison results are shown in Fig. 1. We can see that our method can correctly identify more essential proteins than the other methods.

**Fig. 2:**
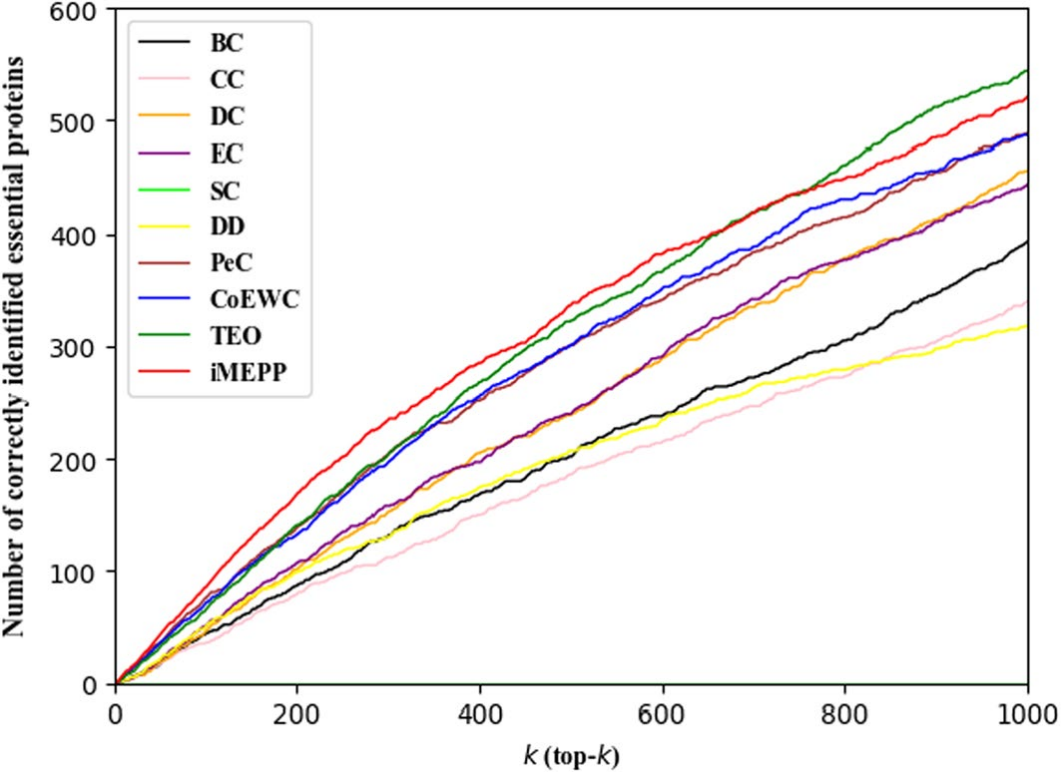
Comparison results when top-*k* (*k* is from 1 to 1000) candidates are output

Figure 2 illustrates the comparison results in a large scale of *k* value: from top-1 to top-1000. We can see that when $k < 667$, our method clearly outperforms the other methods. And when *k* falls in [667, 764], our method performs similarly to TEO. However, when $k > 764$, TEO surpass our method, and our method lies in the 2nd place in these methods.

## Discussion

PIN based computational methods have achieved great success in essential protein identification. Due to the similarity of topological property between PINs and social networks, the IM mechanism of social network is applied to PINs, and then the iMEPP method is proposed to identify essential proteins. First, the PPI data, gene expression data and GO are collected to construct weighted PINs. Then, by using PIN topology and protein orthology, the IS of each protein is calculated to quantify the probability that it is an essential protein. Finally, an ID algorithm is designed to enumerate the candidate essential proteins one by one in an iterative way. Though experimental results on *saccharomyces cerevisiae* data set have shown the effectiveness of the iMEPP method, and its advantage over the existing computational methods, there are still some possible improvements on the method. On the one hand, in iMEPP only one essential protein candidate is identified in each iteration, and totally *k* iterations are done to mine all *k* essential protein candidates. In other words, the time complexity $O(k*|V|+|E|)$ is related to the number *k* of iterations. It is possible to reduce the iteration number by selecting more than one essential protein candidate in each iteration. Therefore, we can speed up the method while maintaining its performance. On the other hand, in social network filed, there are a number of impact maximization algorithms, we are considering to adopt more advanced IM methods to boost essential protein identification from PINs. Furthermore, we will apply iMEPP to the PIN data of other species to identify essential proteins to demonstrate its applicability.

## Conclusion

This paper introduces a novel method for identifying essential proteins from PINs based on IM, which was originally used in social networks for viral marketing. To this end, we define the influence score for nodes in PINs with both orthology data and PIN topological information, and devise an influence discount algorithm to identify essential proteins from PINs. Furthermore, we combine PPI data, gene expression data and GO to construct weighted PINs, which can effectively enhance the quality of PINs. Our experimental results show that the iMEPP method outperforms the existing methods, which demonstrates its effectiveness and advantage.

## Methods

In this section, we present the iMEPP method to identify essential proteins from PINs. First, we introduce the basic concepts of IM, and then give an overview of the iMEPP method. Following that, we give the technical details of the proposed method. Finally, we present the algorithm and the complexity analysis.

### Preliminaries

IM is an important and extensively studied algorithmic problem in social networks, originally motivated by viral marketing [25]. Essentially, it is to select a small number of seed nodes from a social network such that the selected nodes can spread their influence to as many other nodes as possible in the network. Up to now, a large number of algorithms have been proposed for the IM problem, such as greedy algorithms [23] and DD algorithms [24] etc.

#### Definition of influence maximization

A social network can be modeled as a weighted graph $G=(V, E)$, where *V* is the set of individuals (users) regarded as nodes, *E* is the set of connections between individuals (users) regarded as edges and each edge is associated with a weight. Influence spreads in the network based on a stochastic cascade model. There are three types of cascade models: 1) the independent cascade model [23], 2) the linear threshold cascade model, and 3) the weighted cascade model.

Given the social network $G=(V, E)$, a influence cascade model and a number *k* of nodes, the problem of IM is to find *k* nodes from the network such that the expected number of nodes influenced by the *k* selected nodes is as large as possible in terms of the influence cascade model. Here, the *k* nodes are regarded as *k* seeds, and the expected number of nodes influenced by the *k* nodes is regarded as influence spread.

#### Degree discount algorithm

Here, we give a brief introduction to the degree discount (DD) algorithm, which is a typical IM algorithm and will be used in this paper. Generally, some greedy algorithms directly use degree to represent the influence of nodes, and tend to select nodes with the largest degree. Unlike these greedy algorithms, the DD algorithm will re-calculate the degrees of neighbors of a new seed node by a discount in each iteration.

Given the set of seed nodes already selected, in order to find a new seed node from the graph *G*, we first generate a subgraph of *G* without the seed set and the edges associated with the seeds, and then recalculate the degrees of nodes in the subgraph. Note that for these nodes that are not the neighbors of seeds, their degrees keep unchanged. That is, we re-calculate only the degrees of the neighbors of seeds. Suppose *u* is a seed node and *v* is a neighbor of *u* in the subgraph. we discount the degree of *v* by 1 intuitively. Actually, degree discount is not done so simply. Instead, it depends on the influence spread model and is modeled as an optimization problem.

### Overview of the iMEPP method

**Fig. 3:**
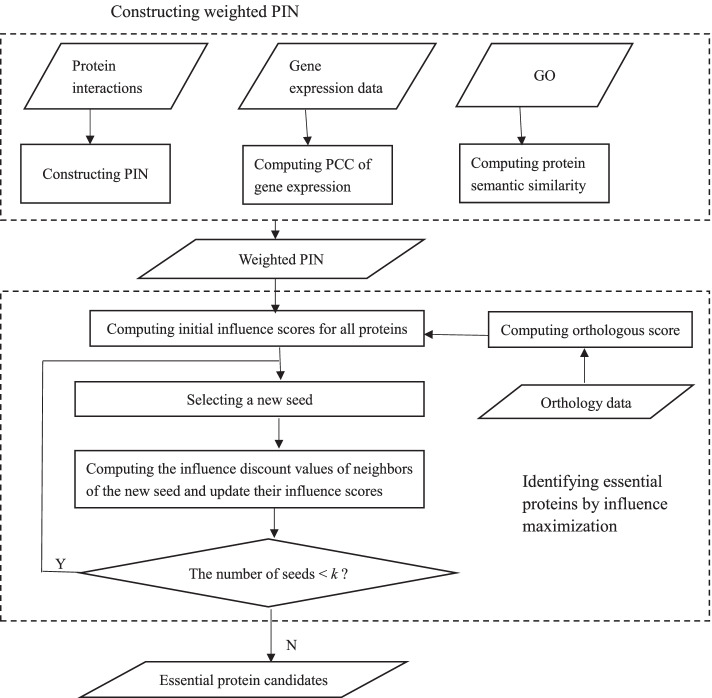
The workflow of iMEPP

Figure 3 shows the workflow of the iMEPP method. It consists of two major modules: weighted PIN construction (in the top dashed-rectangle) and essential protein identification by IM (in the bottom dashed-rectangle).

To construct the weighted PIN, we use PPI data, gene expression data and GO. The PIN edges are weighted by PCC of gene expression and *GO semantic similarity*.

To identify essential proteins by IM, we first compute the initial IS of all proteins in the PIN. The initial IS value of each protein consists of two parts: one is derived from its orthological information, the other is derived from the weights of its connecting edges. Then, we enumerate the essential protein candidates one by one in an iterative way. In each iteration, there are three major steps:

1. Select a new seed $s_{new}$ with the largest IS value from the current remaining proteins (these do not include the nodes in seed set)
2. Compute the *influence discount* (ID) of the non-seed neighbors of $s_{new}$, and update their IS values
3. Check whether the number of selected seeds reaches the desirable value (say *k*). If no, go to next iteration; Otherwise, the iteration is ended and all selected seeds are output as essential protein candidates.

In the following subsection, we will introduce the technical details of the process of identifying essential protein candidates by IM.

### Technical details

Given the original PIN *G*(*V*, *E*), gene expression data, GO and orthology data, we first describe how to construct the weighted PIN, and then introduce how to evaluate the IS and the ID of a protein in the network.

#### Weighted PIN construction

To enhance the quality of PINs and thus to boost essential protein identification accuracy, we construct weighted PINs with gene expression data and GO. Given two proteins *u* and *v*, their corresponding gene expression profiles $p_u$ and $p_v$, we use Pearson correlation coefficient (PCC) [26] to evaluate the level of gene co-expression of *u* and *v* as follows:

1 $$\begin{aligned} PCC(u,v) = \frac{1}{m-1} \sum _{i=1}^{m} \frac{p_{u}(i)-\bar{p}_{u}}{\sigma _{u}} \frac{p_{v}(i)-\bar{p}_{v}}{\sigma {v}}, \end{aligned}$$

where *m* is the number of sampling points of gene expression profiles, $p_{u}(i)$ and $p_{v}(i)$ indicate the gene expression levels at the *i*-th sampling point of proteins *u* and *v* respectively, $\bar{p}_{u}$ and $\bar{p}_{v}$ are the corresponding average values of expression levels, $\sigma _{u}$ and $\sigma _{v}$ are the corresponding standard deviations.

We then calculate the *semantic similarity* of two proteins *u* and *v* by GO. A protein is usually annotated by several GO terms, and the *semantic similarity* between proteins *u* and *v* is calculated as

2 $$\begin{aligned} Sim_{GO}(u, v)=\frac{\sum \limits _{1\le i\le m}Sim_{GO}(t_{u}^{i},v) +\sum \limits _{1\le j\le n}Sim_{GO}(t_{v}^{j},u)}{m+n} \end{aligned}$$

where *u* and *v* are annotated by *m* GO terms $\{t_{u}^{i}|i=1, \ldots , m\}$ and *n* GO terms $\{t_{v}^{j}|j=1, \ldots , n\}$ respectively. $Sim_{GO}$(*t*, *P*) is the *semantic similarity* between GO term *t* and protein *P* annotated by *k* terms:

3 $$\begin{aligned} Sim_{GO}(t, P)=\max _{1\le i\le k}(Sim_{GO}(t, t_{P}^{i})). \end{aligned}$$

Above, the *semantic similarity* of two GO terms $t_1$ and $t_2$ is as follows:

4 $$\begin{aligned} Sim_{GO}(t_{1},t_2) = \frac{\sum _{t \in T_{t_{1}} \bigcap T_{t_2}}(S_{t_{1}}(t) + S_{t_2}(t))}{\sum _{t \in T_{t_{1}}} S_{t_{1}}(t) + \sum _{t \in T_{t_2}} S_{t_2}(t) }, \end{aligned}$$

where $T_{t_{1}}$ (or $T_{t_2}$) is the set of ancestor GO terms of GO term $t_{1}$ (or $t_2$) and itself, and $S_{t_{1}}(t)$ (or $S_{t_2}(t)$) is the *S*-value [27] of GO term *t* related to $t_{1}$ (or $t_{2}$).

The *weight* of the edge connecting *u* and *v* is evaluated as

5 $$\begin{aligned} w(u,v) = Sim_{GO}(u,v) * PCC(u,v), \end{aligned}$$

which measures the association degree of two proteins in the PIN.

#### Influence score (IS)

The influence of a node in a network means its importance in the network. In our scenario, the IS of a protein indicates the probability that it is an essential protein. We consider this from two perspectives: PIN topology and protein orthology.

From the perspective of PIN topology, the IS of protein *u* is as follows:

6 $$\begin{aligned} IS_{topo}(u) =\frac{Inf_{topo}(u)}{\max \{Inf_{topo}(v)|v \in V\}}, \end{aligned}$$

where $Inf_{topo}(u)$=$\sum _{v \in N_{u}} w(u,v)$, $N_{u}$ is the set of neighbors of *u*.

From the perspective of protein orthology, essential proteins usually have orthologs in more species than non-essential proteins. So the orthologous score (OS) [28] can be used to measure the essentiality of proteins. For protein *u*, *OS*(*u*) = $n_{u}$/*N* where $n_{u}$ is the number of species that protein *u* has orthologs and *N* is the total number of reference species. Actually, we use normalized OS to measure the IS of a protein from orthology perspective. That is,

7 $$\begin{aligned} IS_{OS}(u) = \frac{OS(u)}{\max \{OS(v)| v \in V\}}. \end{aligned}$$

Combining $IS_{topo}$ and $IS_{OS}$, the IS of protein *u* is evaluated as follows:

8 $$\begin{aligned} IS(u) = \lambda * IS_{OS}(u) + (1-\lambda ) * IS_{topo}(u), \end{aligned}$$

where $\lambda$ is a tradeoff parameter in [0, 1] to balance the contribution of topology and orthology.

#### Influence discount (ID)

When a protein is selected as seed, the influences of neighbors of this new seed will be discounted and updated. Note that 1) discount is performed only on the topological part of IS as only this part is related to the interaction between proteins. 2) The discount operation depends on the employed influence spreading model. Here, we use the independent cascade model. 3) In each iteration, the discount operation on a protein is performed independently from those performed on it in the previous iterations, which considers all its seed neighbors up to the current iteration. We give the following theorem to indicate how to calculate the ID of a protein.

##### Theorem 1

Given protein *v*, *N*(*v*) is its neighbors set, *t*(*v*) is the number of seed nodes in *N*(*v*), *tt*(*v*) is the sum of *weights* of edges connecting *v* and the seed nodes in *N*(*v*), and *Star*(*v*) is a subgraph consisting of all nodes in *N*(*v*) and the edges connecting to *v*. Under the independent cascade model with spread probability *p*, suppose the following equations hold:

9 $$\begin{aligned} Inf_{topo}(v) = O(1/p),\ tt(v) = O(1/p), \ t(v) = o(1/p). \end{aligned}$$

The influence discount of *v*, denoted by *ID*(*v*), is the expected value of influence of node *v*, derived from the topological information between *v* and the non-seed nodes in *Star*(*v*). Formally,

10 $$\begin{aligned} ID(v) = (Inf_{topo}(v) - tt(v) - (Inf_{topo}(v) - tt(v)) * t(v)*p) * p. \end{aligned}$$

##### Proof

The node *v* is not influenced by any seed node in *N*(*v*) with probability $(1-p)^{t(v)}$. With the spread probability *p*, the value of influence of node *v* generating from the weights between *v* and the non-seed nodes in *Star*(*v*) is $(Inf_{topo}(v) - tt(v)) * p$. Thus, the ID of node *v* is $(1-p)^{t(v)} * (Inf_{topo}(v) - tt(v)) * p$. It derives that 

$$\begin{aligned} ID\left( v \right) & = (1 - p)^{{t(v)}} *(Inf_{{topo}} (v) - tt(v))*p \\ & = (1 - t(v)*p + o(t(v)*p))*(Inf_{{topo}} (v) - tt(v))*p \\ & = [Inf_{{topo}} (v) - tt(v) - (Inf_{{topo}} (v) - tt(v))*t(v)*p]*p + o(t(v)*p) \\ & = [Inf_{{topo}} (v) - tt(v) - (Inf_{{topo}} (v) - tt(v))*t(v)*p + o(t(v))]*p \\ & = [Inf_{{topo}} (v) - tt(v) - (Inf_{{topo}} (v) - tt(v))*t(v)*p]*p. \\ \end{aligned}$$

 Above, the second equality is valid due to the equation $t(v)* p = o(1)$, the third equality holds due to the equation $(Inf_{topo}(v) - tt(v)) * p = O(Inf_{topo}(v) * p) = O(1)$, and the last equality is valid because of the equation $t(v) = o(1/p)$. $\square$

Note that we can guarantee the three equations in Eq. (9) to hold by setting a small value of *p* in experiments. According to Theorem 1, we conclude that the IS of protein *v* in topology is updated as follows:

$$\begin{aligned} IS_{topo}(v) = \frac{ID(v)/p}{\max \{ Inf_{topo}(u) | u \in V \}}. \end{aligned}$$

### Algorithm

Algorithm 1 outlines the procedure of iMEPP. Line 1 initializes the set of essential protein candidates and the parameters. Lines 2–8 compute the initial IS values for all proteins in the PIN, among which Lines 3–5 evaluate the weight between any two interacting proteins. Line 9 gets the maximal value of $Inf_{topo}$. Lines 10–19 cover the iterative process of selecting seeds: Line 11 selects a new seed $s_{new}$ with the largest IS, Line 12 updates the seed set, and Lines 13–18 are for computing the ID values for the non-seed neighbors of $s_{new}$, and updating their IS values. Line 20 returns the seed set as essential protein candidates.
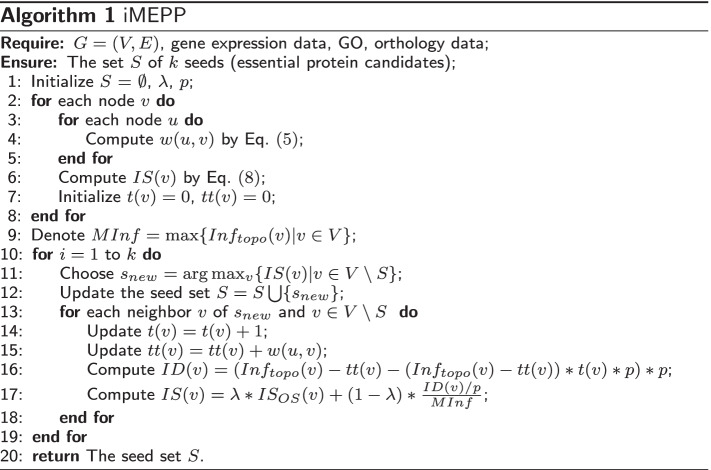


#### Complexity analysis

The time complexity of iMEPP consists of two parts. The first part is the calculation of initial *IS* values for all proteins in a PIN, which is totally determined by the number of edges. Thus, the time complexity of this part is *O*(|*E*|). The second part is related to the iterative procedure of seed selection. The time complexity for each iteration is $O(\log |V|)$. Therefore, the time complexity of the second part is $O(k * \log |V|)$. In summary, the complexity of iMEPP is $O(k * \log |V| + |E| )$.

## Data Availability

The datasets used and/or analysed in this study are available in the corresponding articles. The source code and data of iMEPP are available at https://github.com/xuweixia88/iMEPP.git.

## Abbreviations

PPI: Protein–protein interaction
PIN: Protein–protein interaction network
GO: Gene ontology
iMEPP: Influence maximization for essential protein prediction
RNA: Ribonucleic acid
DC: Degree centrality
BC: Betweenness centrality
CC: Closeness centrality
SC: Subgraph centrality
EC: Eigenvector centrality
PCC: Pearson correlation coefficient
BioGRID: Biological General Repository for Interaction Datasets
SGD: Saccharomyces genome database
DEG: Database of essential genes
DD: Degree discount
IM: Influence maximization
IS: Influence score
ID: Influence discount
OS: Orthologous score
